# A Proinflammatory Immune Response Might Determine *Toxoplasma gondii* Vertical Transmission and Severity of Clinical Features in Congenitally Infected Newborns

**DOI:** 10.3389/fimmu.2020.00390

**Published:** 2020-03-13

**Authors:** Fernando Gómez-Chávez, Irma Cañedo-Solares, Luz Belinda Ortiz-Alegría, Yevel Flores-García, Ricardo Figueroa-Damián, Héctor Luna-Pastén, Valeria Gómez-Toscano, Carlos López-Candiani, G. Emmanuel Arce-Estrada, Christian A. Bonilla-Ríos, Juan Carlos Mora-González, Ricardo García-Ruiz, Dolores Correa

**Affiliations:** ^1^Instituto Nacional de Pediatría, Secretaría de Salud, Mexico City, Mexico; ^2^Cátedras CONACyT-Instituto Nacional de Pediatría, Mexico City, Mexico; ^3^Departamento de Formación Básica Disciplinaria, ENMyH-IPN, Mexico City, Mexico; ^4^Instituto Nacional de Perinatología, Mexico City, Mexico; ^5^Centro de Salud Gustavo A. Rovirosa, Mexico City, Mexico; ^6^Laboratorio de Cannabinoides, Facultad de Medicina, UNAM, Mexico City, Mexico

**Keywords:** *Toxoplasma gondii*, immune response, human congenital toxoplasmosis, vertical transmission, TGF-β1

## Abstract

*Toxoplasma gondii* is the etiological agent of toxoplasmosis. Mother-to-child transmission of this parasite can occur during pregnancy. Newborns with congenital toxoplasmosis may develop central nervous system impairment, with severity ranging from subclinical manifestations to death. A proinflammatory/regulated specific immune profile is crucial in the defense against the parasite; nevertheless, its role in the infected pregnant women and the congenitally infected offspring has been poorly explored, and there is still no consensus about its relation to parasite vertical transmission or to severity and dissemination in the congenitally infected newborns. This work aimed to characterize these relations by means of principal component and principal factor analyses. For this purpose, we determined the specific production of the four immunoglobulin G antibody subclasses, cytokines, and lymphocyte proliferation in the *T. gondii–*infected pregnant women−10 who transmitted the infection to their offspring and seven who did not—as well as in 11 newborns congenitally infected and grouped according to disease severity (five mild and six moderate/severe) and dissemination (four local and seven disseminated). We found that the immune response of nontransmitter women differed from that of the transmitters, the latter having a stronger proinflammatory response, supporting a previous report. We also found that newborns who developed moderate/severe disease presented higher levels of lymphocyte proliferation, particularly of CD8^+^ and CD19^+^ cells, a high proportion of tumor necrosis factor α producers, and reduced expression of the immune modulator transforming growth factor β, as opposed to children who developed mild clinical complications. Our results suggest that a distinctive, not regulated, proinflammatory immune response might favor *T. gondii* vertical transmission and the development of severe clinical manifestations in congenitally infected newborns.

## Introduction

Toxoplasmosis is a worldwide highly prevalent zoonotic infection caused by the parasite *Toxoplasma gondii*, which can be orally or congenitally acquired (1, 2). Humans can become infected *per os* through the ingestion of food or water contaminated with sporulated oocysts released in the feces of the definitive hosts (felines) or of undercooked meat products containing tissue cysts (3), as well as by vertical transmission from mother to fetus during pregnancy (4–6). Congenital toxoplasmosis can promote clinical manifestations in the newborn that involve the central nervous system, such as hydrocephalus and hearing loss, or the eye, such as retinochoroiditis (7–9). Given the high prevalence of this disease and the wide range of clinical manifestations, it is of great importance to study the factors involved in congenital transmission of *T. gondii* and the severity of congenital toxoplasmosis in the newborn, but these aspects have been poorly explored. Both humoral and cell-mediated proinflammatory responses have consistently been reported as crucial for the containment of this parasite in acquired toxoplasmosis (10). Regarding fetal damage, it has been hypothesized that disease results from the poorly developed fetal immune response, which is thought to be unable to control parasite replication (11), allowing *T. gondii* to invade different tissues of the host and thus contributing to the high heterogeneity of the clinical manifestations associated with the infection (9). This has been reinforced by the known inverse relation between the gestation time at infection and the severity degree (4, 12). Nevertheless, contrasting results have suggested that besides parasite virulence a nonregulated exacerbated proinflammatory response that promotes tissue damage may contribute to the development of toxoplasmosis, which has been studied mainly in rodent models, with few studies in humans (5, 13–19). In addition, we and others have reported that the mother's immune response could be involved in the transmission of the parasite through the placenta during pregnancy (10, 18, 19). The major limitation in the study of human congenital toxoplasmosis regards to interspecies differences, since the insights gained from mice models have not been tested for fidelity in humans (16–19); that is, the central processes that occurred in the placenta in humans can mainly be studied at the culmination of pregnancy, when transmission might have already happened. To evaluate the role of the maternal immune system during pregnancy and parturition in *T. gondii* transmission, we have evaluated systemic cellular and humoral-specific response against the parasite and after pregnancy in the infected newborns; interestingly, we found that some immunoglobulin G (IgG) subclasses might be used as markers of transmission of newborns' poor prognosis and that a nonregulated proinflammatory response in the infected mother might be related to parasite vertical transmission (19). Nevertheless, our previous approaches were limited in the number of immunological markers in a reduced group of patients, and they were not analyzed to define which of these conform a profile in transmitters (19). Even more, the role of the infected fetus immune response in the dissemination and severity of congenital toxoplasmosis has not been analyzed.

In this study, we aimed to determine the role of maternal immunological profile in transmission and that of the congenitally infected fetus/newborn on disease dissemination or severity.

## Materials and Methods

### Ethical Aspects

This work was performed according to the World Medical Association's Declaration of Helsinki. The project (INP 060/2011) was approved by the Research and Research Ethics Boards of the Instituto Nacional de Pediatría (INP), Mexico City, Mexico. This institutional review boards (IRBs) are registered at the Office for Human Research Protection of the National Institutes of Health (http://ohrp.cit.nih.gov/search/search.aspx) with numbers IRB00008064 and IRB00008065. Institutional review board approvals are available upon request. The Instituto Nacional de Perinatología (INPer) IRBs also approved the project (212250-02231). All participant pregnant women and/or tutors of infected children signed corresponding informed consents, in which we explicitly stated that it was of low risk, considering that clinical management would not be modified for the protocol.

### Patients and Study Strategy

All pregnant women and newborns were, respectively, managed at INPer/“Centro de Salud-Dr. Gustavo A. Rovirosa Pérez” in Mexico City and INP, according to national and international standards. For this study, we included 17 pregnant women who were diagnosed as positive for *T. gondii* infection and had no other disease. As specified in Supplementary Table 1, data from 7/17 infected pregnant women were taken from Gómez-Chávez et al. (19) to reanalyze them by the methodology described herein. Mothers were considered at risk of vertical transmission during pregnancy, by the presence of low-avidity IgG antibodies, *T. gondii* polymerase chain reaction (PCR)–positive results, or clinical evidence or presence of IgM/IgG antibodies concomitant with clinical history of obstetric diseases suggestive of toxoplasmosis (such as recurrent abortions or previous premature or stillbirth deliveries). To classify women at risk in nontransmitters or transmitters (Supplementary Table 1), we followed their gestations until delivery and the surviving children for at least 1 year (9). We confirmed 11 congenitally infected newborns (0–65 days of life), seven from prenatally screened mothers of this study and four who arrived as clinical cases to INP. Four newborns of the studied transmitter mothers could not be recruited for immunological tests because they died soon after confirmation or they were not brought for continuation of treatment.

Confirmation of congenital infections was done through serological tests by the detection of specific IgM and IgG neoantibodies by enzyme-linked immunosorbent assay (ELISA) and Western blot and through B1 parasite gene detection by quantitative PCR on peripheral blood, according to previously reported methods and algorithms (9, 19–22). After the infection was confirmed and before treatment onset, we determined the levels of specific anti–*T. gondii* IgG1, IgG2, IgG3, and IgG4 antibodies and proliferation *in vitro* of peripheral blood mononuclear–specific cells and their cytokine production (19). Congenitally infected newborns were evaluated at 1-year follow-up, and their clinical features classified according to the location (neurological or disseminated) and severity (mild or moderate/severe) (9). In brief, local infection refers to patients with neurological, ophthalmic, or both alterations, such as intracranial calcifications, hydrocephalus, microcephalus, retinochoroiditis, uveitis, and intraocular hemorrhage. The infection was considered disseminated when the patients had local clinical manifestations and at least one of the following: low birth weight, hepatomegaly or cholestasis, splenomegaly, intrahepatic calcifications, petechiae rash eruptions, myocarditis, anemia, thrombocytopenia, eosinophilia, or pneumonitis. Congenital toxoplasmosis patients were assigned to the mild disease group if they were subclinical during physical examination, showed isolated findings, or responded adequately to treatment, as determined through follow-up of the disease clinical presentation in which we assessed the children's weight and height, as well as their auditive, muscular, neurological, and ophthalmological development. Children in this group showed diminished lesions in the affected organs or a limited development of severe clinical manifestations, expected on the basis of the evolution before treatment and the untreated cases reported in the literature. In contrast, patients were classified as having a severe disease if they presented clinical manifestations that compromised their life or because they did not respond appropriately to treatment (Supplementary Table 2).

### Anti–*T. gondii* Antibody Detection

Detection of serum anti–*T. gondii* IgG1, IgG2 IgG3, and IgG4 antibodies was performed using indirect homemade ELISAs (10, 19, 21). Subclasses were detected using biotinylated monoclonal antibodies against each IgG subclass: IgG1 (clone 8c/6-39), IgG2 (clone HP-6014), IgG3 (clone HP-6050), and IgG4 (clone HP6025) (Sigma Chemical Co., St. Louis, MO, USA). Polystyrene plates (Maxisorp, Nunc, Roskilde, Denmark) were coated with 5 μg/mL of the RH strain crude extract of *T. gondii*, blocked with 1% bovine serum albumin–phosphate-buffered saline (PBS), and incubated with serum diluted in PBS−0.05% Tween 20, to 1:250 for IgG1, and 1:125 for IgG2 to IgG4. The reaction was developed with horseradish peroxidase–streptavidin (Sigma Chemical Co.) diluted 1:10,000. The enzymatic activity was revealed with *O*-phenylenediamine/H_2_O_2_, in citrate buffer (pH 5.0) and stopped with 0.1 N sulfuric acid. The absorbance was read at 492 nm. The results were expressed as the reactivity index (RI), which was calculated by dividing the mean absorbance of duplicates of each sample by the cutoff value for each immunoglobulin (mean plus 3 standard deviations of six low, medium, and high negative controls). An RI ≥ 1.0 was considered positive (21).

### Peripheral Blood Samples and Proliferation Test

Five milliliters of peripheral blood from pregnant women at risk of vertical transmission and congenitally infected newborns was obtained into EDTA-Vacutainer tubes. To obtain peripheral blood mononuclear cells (PBMCs), peripheral blood was subjected to Ficoll-Hypaque separation (GE Healthcare, Piscataway, NJ, USA). Then, PBMCs were labeled with carboxyfluorescein succinimidyl ester (CFSE) (Molecular Probes/Invitrogen Detection Technologies, Eugene, OR) as previously reported (19). Briefly, cells were stimulated with 10 μg/mL of the soluble *T. gondii* antigen (STAg) in triplicate and then incubated at 37°C in 5% CO_2_/95% air atmosphere, for 72 h for cytokine detection and 120 h for cell proliferation assays. Peripheral blood mononuclear cells cultured without STAg stimulation were used as negative controls. We used the mitogen ConA at 5 μg/mL as positive response ability control. After the stimulation period, CFSE-labeled PBMCs were collected and stained with the following antibodies: CD3/APC-Cy7 (SK7), CD4/APC(OKT4), CD8/PERCP-Cy5 (RPA-T8), and CD19/PE-Cy7 (HIB19) (BioLegend, San Diego, CA, USA). All samples were analyzed in a FACS-Aria flow cytometer (BD Biosciences, San Jose, CA, USA) using FlowJo (version 7) software (Tree Star Inc., Ashland, OR, USA). For the analysis, 1 × 10^4^ events were collected. Relative proliferation index was calculated by dividing the proliferation percentage of STAg-stimulated PBMCs by the proliferation percentage of nonstimulated PBMCs. A proliferation index ≥ 1.0 was considered positive.

### Cytokine Detection in PBMC Culture Supernatants

The concentration of interleukin 1β (IL-1β), IL-2, IL-4, IL-5, IL-6, IL-8, IL-10, IL-12p70, tumor necrosis factor α (TNF-α), interferon γ (IFN-γ), and transforming growth factor β1 (TGF-β1) in the supernatants obtained from PBMC cultures were quantified using the human inflammatory CBA kit, the human T_H_1/T_H_2 CBA kit, and the human TGF-β1 Single Plex Flex set (BD Biosciences). The CBA analysis was performed with the FCAP Array Software v3.0 (BD Biosciences), from data obtained in a BD FACSAria flow cytometer (BD Biosciences). Cytokine production index for each cytokine tested was calculated, dividing cytokine concentration from STAg-stimulated PBMCs by the cytokine concentration of the negative (only medium) PBMC controls. A cytokine production index ≥ 1.0 was considered positive.

### Data Analysis and Statistics

The standardized data of the levels of 18 immunological markers (IgG1, IgG2 IgG3, and IgG4; IL-1β, IL-2, IL-4, IL-5, IL-6, IL-8, IL-10, TNF-α, and IFN-γ, as well as total lymphocytes, CD3^+^, CD4^+^, CD8^+^, and CD19^+^) from nontransmitters, transmitters, and congenitally infected newborns were subjected to a principal component analysis (PCA). We maximized the variance of the normalized loadings from the principal components by the varimax rotation and retained as signifiers each component associated with a singular value of at least 1.0 and with at least 10% of the total variance. We extracted the underlying principal common factors by estimation of commonalities from the orthonormalized loadings of the retained components. This multivariate analysis was done on MATLAB 2018a version (23). On the other hand, the antibody RI, the cytokine production index, and the proliferation index were compared between congenitally infected newborns with local vs. disseminated and mild vs. moderate/severe disease, determining the statistical significance using the Mann-Whitney *U* test and comparing proportions between groups using the Fisher exact test. A *p* ≤ 0.05 was considered significant. The GraphPad Prism version 7.0d for Macintosh (GraphPad Software, La Jolla, CA, USA; www.graphpad.com) was used for these purposes.

## Results

We evaluated 20 immunological markers in 10 *T. gondii* transmitter and seven nontransmitter women, as well as in 11 newborns with congenital toxoplasmosis. Interleukin 12 was not expressed by any patient and thus was excluded from the PCA. Transforming growth factor β was also excluded because of the reduced number of tested samples in the infected pregnant women group, resulting in an unequal representation for PCA. The multivariate analysis showed a specific pattern of the molecular markers in each group, and the nontransmitter women, the transmitter women, and the congenitally infected newborns were contained in four PCs (Table 1). As it is shown, nontransmitter women can be described by nine of the 18 markers, which account for 93.2% of the total variance, principal component 1 (PC1): ↑TNF-α and ↑IL-2; PC2: ↑IgG3 and ↓IL-8; PC3: ↑IgG1, ↑IgG2, and ↑IgG4; PC4: ↑CD8 and ↓IL-4. More heterogeneous results were found among transmitter women, because seven markers sum up the group, representing 76.8% of the total variance, PC1: ↑TNF-α and ↑IFN-γ; PC2: ↑IgG4; PC3: ↑IL-6 and ↑IL-8; PC4: ↑CD19 and ↑Lym (lymphocytes proliferation). Remarkably, TNF-α had a similar vectoral effect between transmitter and nontransmitter groups, whereas IL-8 had an opposed result.

**Table 1:** Principal component analysis from 18 molecular markers and their rotated loadings.

	**Non-transmitter women**				**Transmitter women**				**Congenitally infected newborns**			
**Principal component**	**1**	**2**	**3**	**4**	**1**	**2**	**3**	**4**	**1**	**2**	**3**	**4**
Singular value	8.97	3.40	2.51	1.90	5.37	3.54	2.87	2.03	5.30	4.32	3.32	1.87
Variance explained (%)	49.82	18.87	13.92	10.55	29.84	19.68	15.95	11.28	29.45	23.99	18.44	10.40
Cumulated variance (%)	49.82	68.70	82.62	93.18	29.84	49.53	65.49	76.77	29.45	53.45	71.89	82.30
**Loadings by molecular marker**												
IgG1	−0.02	0.05	**0.59**	0.02	0.34	0.18	0.03	0.35	−0.13	0.32	0.02	0.07
IgG2	0.19	−0.20	**0.47**	0.22	−0.05	0.04	0.04	−0.10	−0.23	0.07	0.38	0.02
IgG3	−0.05	**0.74**	0.00	0.16	0.33	−0.01	−0.11	0.01	−0.08	**0.67**	0.02	−0.13
IgG4	0.04	0.14	**0.47**	−0.26	0.13	**0.69**	0.08	0.08	0.06	0.20	**0.55**	−0.04
CD3	0.39	0.00	0.01	0.01	0.13	−0.16	0.02	0.12	0.00	0.02	−0.03	**0.67**
CD4	0.20	0.04	−0.01	0.04	−0.13	0.16	−0.03	−0.04	−0.03	−0.04	0.01	**0.71**
CDS	0.11	−0.01	0.12	**0.56**	0.17	−0.35	0.04	0.09	0.01	−0.01	−0.11	0.00
CD19	0.08	−0.05	0.06	−0.08	−0.13	0.26	−0.08	**0.48**	0.16	**0.50**	0.02	0.12
Lym	−0.31	0.12	0.19	0.37	−0.07	−0.08	−0.03	**0.71**	**0.59**	0.16	0.05	0.09
IL-1	0.29	0.06	0.00	0.05	0.20	−0.03	−0.12	−0.10	0.02	−0.02	0.00	0.01
IL-2	**0.40**	0.00	−0.01	−0.01	0.25	−0.28	0.22	0.18	0.02	0.11	−0.02	0.04
IL-4	−0.06	−0.04	0.33	**−0.51**	−0.13	−0.27	0.03	0.12	0.18	−0.02	−0.01	0.01
IL-5	0.17	−0.05	−0.07	0.01	0.08	0.11	−0.03	−0.04	**0.50**	−0.04	0.02	−0.06
IL-6	0.38	−0.00	0.07	0.09	0.06	−0.12	**0.66**	0.02	0.09	−0.19	**0.58**	0.01
IL-8	−0.12	**−0.60**	−0.01	0.14	−0.09	0.20	**0.67**	−0.06	−0.04	−0.15	**0.45**	0.03
IL-10	−0.21	−0.05	0.14	−0.01	0.25	0.01	−0.04	−0.19	−0.05	0.01	−0.03	0.03
TNFα	**0.41**	0.02	0.00	−0.02	**0.48**	−0.05	0.04	−0.01	**0.48**	−0.05	0.01	−0.02
IFNγ	0.09	0.07	−0.11	−0.32	**0.49**	0.07	−0.05	−0.07	0.10	−0.20	−0.04	−0.07

*We show the first four significant components (with a singular value > 1, and a total explained variance > 10% each). The correlation coefficient of the components was significant (bold) as the varimax rotated loadings increase, from a relatively weak relationship (0.4–0.5) to the middle (0.5–0.7) or strongest (>0.7)*.

The response of the congenitally infected newborns is composed by 10 markers that concentrate 82.3% of the total variance (PC1: ↑Lym, ↑IL-5, and ↑TNF-α; PC2: ↑IgG3 and ↑CD19; PC3: ↑IgG4, ↑IL-6, and ↑IL-8; PC4: ↑CD3 and ↑CD4): three unique to this group (↑CD3, ↑CD4, and ↑IL-5), four shared with transmitter women (↑TNF-α, ↑Lym, ↑IL-6, and ↑IL-8), and three with nontransmitter women (↑TNF-α, ↑IgG3, and ↑IgG4) (Table 1). Finally, ↑TNF-α was shared by all groups and all in the first component. Then, a varimax rotation of scores analysis was performed, and we extracted principal common factors for each group from a four-factor solution. For nontransmitter women, we found that factor 2 is the central common factor and includes IgG3 in high commonality with low production of IL-8. The group of transmitter women is described by the principal common factor 2, represented by IgG4; common factor 3, with IL-8 in high commonality with IL-6, and common factor 4, with Lym in high commonality with CD19.

**Figure 1:**
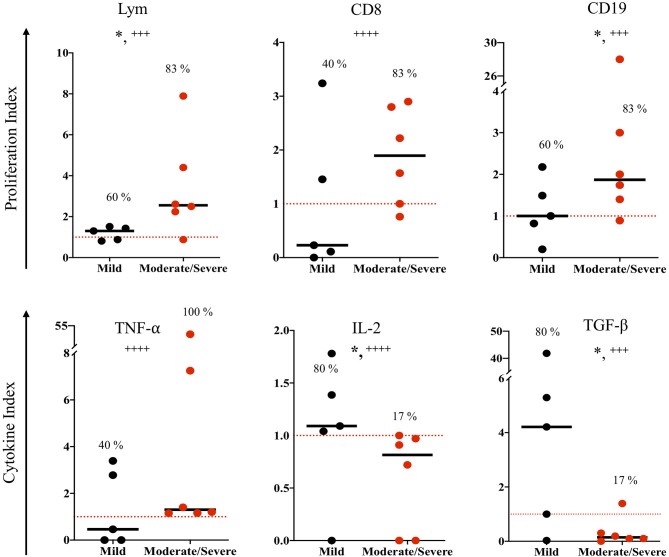
Immune response to soluble *Toxoplasma gondii* antigen (STAg) of congenitally infected newborns according to severity clinical manifestations. The horizontal line indicates the median; each dot represents the result of one individual. The red dashed lines show the cutoffs. Mild vs. moderate/severe clinical groups were classified according the criteria previously reported (9). Mann-Whitney *U* test, *p ≤ 0.05; Fisher exact test, + + ++*p* ≤ 0.0001, + + +*p* ≤ 0.001.

**Figure 2:**
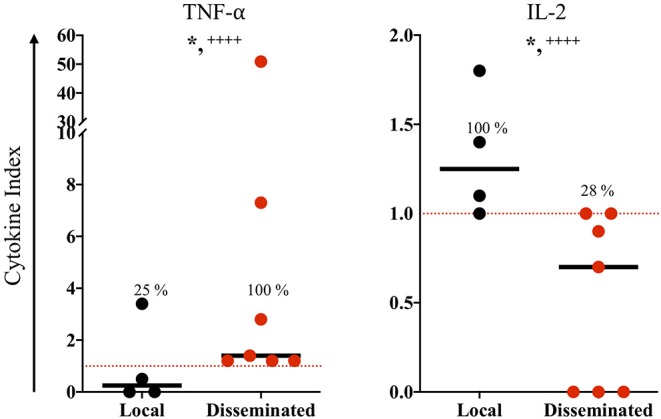
*In vitro* immune response of congenitally infected newborns according to disease dissemination. The horizontal line indicates the median; each dot represents the result of one individual. The red dashed lines show the cutoffs. Mild vs. moderate/sever clinical groups were classified according the criteria reported previously (9). Mann-Whitney *U* test, *p ≤ 0.05; Fisher exact test, + + ++*p* ≤ 0.0001.

**Figure 3:**
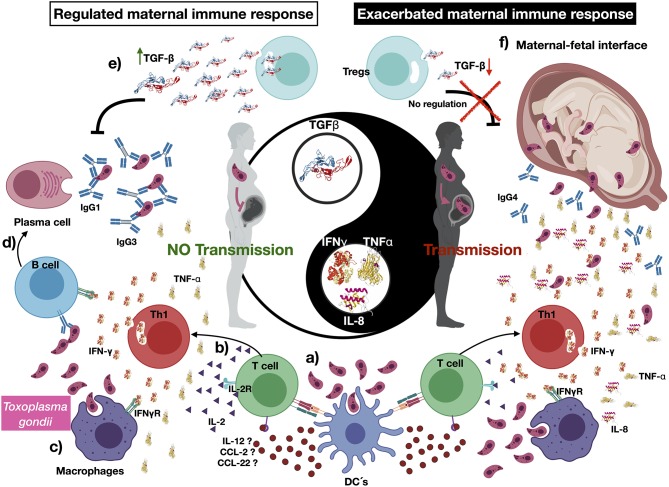
The immune response in congenital transmission, an immune-regulated maternal response limit *Toxoplasma gondii* congenital transmission. (a) *Toxoplasma gondii* may be recognized by pregnant women innate immune cells, such as dendritic cells (DCs); in turn, they can phagocyte, process parasite antigens, and produce cytokines and chemokines such as interleukin 12 (IL-12), CCL-2, and CCL-22, which activate naive T cells. (b) Once naive T cells are activated by the recognition of *T. gondii* antigens, they produce cytokines such as IL-2, interferon γ, and tumor necrosis factor α. (c) The development of such a proinflammatory environment stimulates other immune cells such as macrophages, which mediate parasite phagocytosis. (d) Several cytokines stimulate the activation of B cells and induce immunoglobulin class switch. (e) We suggest that transforming growth factor β (TGF- β) may have a pivotal role in controlling *T. gondii* vertical transmission, severity, and dissemination of the congenital infection. (f) A reduced production—or absence—of TGF- β relates to an exacerbated proinflammatory profile, which may be associated to vertical transmission and the development of severe and disseminated disease in the *T. gondii*–infected newborns. Created with BioRender.com.

The congenitally infected newborns are represented by the principal common factor 4, containing CD4 proliferation in high commonality with CD3 (Table 2). Because of the few cases in this group, we could not compare common factors between subgroups. But, because the immunological response may be involved in the development of clinical manifestations in the newborns, we directly compared the immunological markers segregating our data according to the severity and dissemination degree. We found that lymphocyte proliferation, particularly of the CD8^+^ and CD19^+^ subpopulations, was higher and in a significantly larger proportion of the newborns with moderate/severe disease than in those with mild symptomatology (Figure 1). Remarkably, the frequency of TNF-α positive cases was significantly higher in newborns with moderate/severe clinical outcome than in patients with mild illness (Figure 1); conversely, the proportion and levels of the immune regulator cytokines IL-2 and TGF-β were higher in the latter group. No differences between groups were observed regarding specific production of the proinflammatory cytokine IFN-γ (data not shown). We found a pattern of increased TNF-α levels and low production of IL-2 and TGF-β production in the congenitally infected newborns, with disseminated toxoplasmosis and an inverse profile in those with localized lesions (Figure 2). Based on our results, we summarized the two faces of the immune response in the inhibition or promotion of congenital transmission and the clinical outcome of the infected newborn, which is discussed below (Figure 3).

**Table 2:** Factor extraction and variance analysis from a four principal factors solution.

	**Non-transmitter women**			**Transmitter women**			**Congenitally infected newborns**		
	**Molecular marker**	**Commonality**	**Unique**	**Molecular marker**	**Commonality**	**Unique**	**Molecular marker**	**Commonality**	**Unique**
Factor 1	**TNFα**	0.17	**0.82**	**TNFα**	0.23	**0.77**	**TNFα**	0.24	**0.76**
	**IL-2**	0.16	**0.84**	**IFNγ**	0.25	**0.75**	**Lym**	0.38	**0.62**
							**IL-5**	0.25	**0.75**
Factor 2	**IgG3**	**0.57**	0.43	**IgG4**	**0.51**	0.49	**IgG3**	0.48	**0.52**
	**IL-8 (–)**	0.4	**0.6**				**CD19**	0.29	**0.71**
Factor 3	**IgG1**	0.36	**0.64**	**IL-6**	0.46	**0.54**	**IL-6**	0.38	**0.62**
	**IgG2**	0.34	**0.66**	**IL-8**	**0.51**	0.49	**IL-8**	0.23	**0.77**
	**IgG4**	0.31	**0.69**				**IgG4**	0.35	**0.65**
Factor 4	**CD8**	0.35	**0.65**	**CD19**	0.32	**0.68**	**CD3**	0.44	**0.56**
	**IL-4 (–)**	0.37	**0.63**	**Lym**	**0.51**	0.49	**CD4**	**0.5**	0.49

*Variance analysis by the principal factor method. The total variance associated with each of the original variables is expressed as the portion of the variance in communality with other markers, explained by a common factor or the portion of unique variance which has nothing in common with any other variable. The unique factor includes the inherent variability for each variable. (–) negative loading, see Table 1*.

## Discussion

The immune response has been proposed as a critical factor involved in the control of the parasite *T. gondii* in individuals with acquired toxoplasmosis (11, 15–19, 24–26). We previously reported that *T. gondii* vertical transmission might be related to those cases positive to proinflammatory cytokines such as IFN-γ and negative to the immunoregulatory cytokine TGF-β (19). Thus, we hypothesized that the immune system profile could be involved in congenital transmission during pregnancy, and it could be a determinant for the development of clinical features in the congenitally infected newborns (11, 19). To study these phenomena, in the present work we analyzed 18 humoral and cellular immunological markers in 17 infected pregnant women at risk of vertical transmission and 11 congenitally infected newborns, using principal components and a common factor analysis.

The comparison of the immunological components and common factors showed that the cytokine TNF-α was the only marker produced in similar levels by the three groups of this work, but it was concentrated among children with severe disease when this group was subclassified. Tumor necrosis factor α is a proinflammatory cytokine that long ago was reported to play a significant role in stimulating parasite-destructive effector mechanisms, in synergy with IFN-γ (27).

Our findings also showed that IL-12 was not detected in any sample challenged with STAg, an observation that has been previously reported by others in PBMCs from cases of ocular toxoplasmosis (28) and healthy donors (29). These reports contrast those about the murine response, probably due to essential differences in the initial steps of parasite recognition; while in mice it is mostly attributed to the innate sensors TLR-11 and TLR-12, triggering production of high levels of IL-12, recognition of the parasite by a specific receptor has still not been elucidated in humans, who lack the mentioned receptors (30). Interestingly, Safronova et al. (29) showed that human PBMCs infected with *T. gondii* secrete large amounts of CCL-2, CCL-22, and IL-8 but not IL-12, remarking the differences between species, as it has also been reported for the response to bacterial infections, challenging the usual view about the role of IL-12 in the human immune response (29, 31). In this work, nontransmitter women showed an increased IL-2 production. This cytokine has a protective role in murine toxoplasmosis models, increasing survival of infected animals and reducing the number of cysts in the brain, by an increased activity of natural killer and CD8^+^ cells (32, 33). Our results remarked the idea of IL-2 as an important cytokine that participates limiting *T. gondii* transmission in the nontransmitter women.

Principal components and common factor analysis also showed characteristic profiles in the studied groups. These analyses allowed us to identify nine molecular markers in four factors, as a non–transmission-related profile, being factor 2 (↑IgG3 and ↓IL-8) the factor with the highest commonality. Interestingly, IgG3 is induced by a proinflammatory, but regulated, environment due to the presence of IL-10 and TGF-β (34, 35). In murine models of acquired toxoplasmosis, there is evidence suggesting that IgG2b (IgG3 in humans) has a protective role against the parasite (36), by induction of complement and phagocytosis, reducing in this way the parasite burden (37). Regarding IL-8, our results suggest that a low production of this cytokine is related to protection against congenital parasite transfer; this is the first report of such relation, because it was found in the serum of pregnant woman infected with *T. gondii*, but it was not studied in relation to transmission or obstetric problems (38). In this context, different groups have demonstrated that *T. gondii* has the potential to control the production of several cytokines, including IL-8 in cells of the maternal–fetal interface, promoting parasite maintenance and proliferation (39, 40). It is well documented that *T. gondii* produces a protein called TgMIF, an analog to host migration inhibitory factor (MIF); both proteins are potent proinflammatory inducers, which manipulate the host immune response in favor of parasite invasion and persistence (41, 42). It is hypothesized that these molecules stimulate ERK 1/2 phosphorylation (anti-apoptotic pathway), inducing IL-8 production, increasing host cell proliferation and differentiation, and globally favoring parasite proliferation in cells from the fetal–maternal interface (41, 42). In concordance with our results, low IL-8 production was an important marker among the nontransmitter women, whereas high production of this cytokine was associated with mother to the fetus parasite transmission during pregnancy.

Factor 4 in nontransmitter woman was positive to CD8^+^ lymphocytes proliferation and reduced IL-4 production. Long ago, the critical function of the CD8^+^ cytotoxic T cells in the inhibition of parasite replication by killing infected cells and promoting tachyzoite to bradyzoite conversion was recognized (43). The activity of these cells in nontransmitter women might be involved in limiting tachyzoites stage proliferation and dissemination through blood, the most crucial parasite stage and pathway associated to congenital infection (5, 14). The relation of CD8^+^ cell proliferation to nontransmission is consistent with the previous work reported by Prigione et al. (44), who showed that PBMCs from mothers who did not transfer the infection to their fetuses induced the proliferation of CD8^+^ active cytotoxic T cells after stimulation *in vitro* with *T. gondii* antigens. Moreover, *T. gondii*–specific CD8^+^ T cells were described as good producers of IL-2, the immunological marker in nontransmitter women group mentioned previously (33).

In the group of transmitter women, we identified the production of IL-8 in commonality with IL-6, the proliferation of B cells in commonality to total lymphocytes proliferation, and the independent effect of the proinflammatory cytokines, TNF-α and IFN-γ. The control of maternal-fetal transmission of the parasite remains almost unknown, but there is evidence suggesting a dual role of proinflammatory cytokines such as TNF-α and IFN-γ (16). In a murine model, Abou-Bacar et al. showed that if IFN-γ was completely neutralized the parasite load augmented in the maternal peripheral blood, but vertical transmission rate decreased, indicating an enhancing effect of IFN-γ on congenital transmission (16–18). The mechanism of this parasite-enhanced passage has been related to IFN-γ induction of MIF, which in turn induces the expression of intercellular adhesion molecule 1 (ICAM-1) on the surface of the villous syncytiotrophoblast. ICAM-1 is known for its capacity to adhere the parasite MIC2, which favors host cell infection by the parasite (15, 45, 46). On the other hand, there are reports showing that *T. gondii* is more frequently transmitted during the third trimester of pregnancy (4), and it is known that the immune profile varies along gestation (47); subsequently, transmission could be related to the immunological environment dependent on the gestational time. Nevertheless, the transmitter group of this study was composed by 50% of women in first and second trimesters of pregnancy (Supplementary Table 1), which suggests that other factors of the parasite or the maternal/fetal hosts could also be involved, but more research is needed to clarify this question. In summary, the present work and previous findings are consistent with an enhancing effect of a proinflammatory but nonregulated profile on vertical transmission (19).

We found that the response of the congenitally infected newborns shared some PC with the transmitter women, such as the production of the proinflammatory cytokines IL-6 and IL-8, the antibody subclass IgG4, and lymphocyte proliferation. Thus, it seems that immune response profile is similar among acutely infected individuals, regardless of age and transmission pathway. But, the immune response of the newborns with moderate/severe disease presented an increased proliferation of the lymphocyte population and specifically of the CD8^+^ and CD19^+^ subtypes, in comparison to those with mild disease, remarking an active immune response against the parasite that does not necessarily protect them, but on the contrary, that might induce clinical problems. Data obtained from animal models support the notion that several fetal and neonatal pathological findings in humans can be related to both uncontrolled parasite replication and tissue necrosis, mediated by a robust T cell response (48–50). Likewise, Roberts et al. (51) reported an association between the presence of T cells and increased ocular damage in retinal lesions from congenitally infected human fetuses and newborns (51), and more recently, a Brazilian group demonstrated an increased lymphocyte (CD4^+^ and CD8^+^) proliferation, as well as an increased production of the TNF-α in congenitally infected newborns, and particularly in those with early and active ocular lesions (52, 53). Despite the previously mentioned protective effect of cytotoxic cells, it is known that this obligate intracellular parasite can use several mechanisms through which it evades not only cellular immunity, but also redirects the cell-mediated cytotoxicity to its advantage, favoring its dissemination (6); they can promote rapid egress of *T. gondii* parasites from infected cells and tissue necrosis by a mechanism dependent on death receptor ligation. The released parasites are able to infect neighboring tissue cells and recruited leukocytes (54), spreading infection locally and systemically due to the ability of the latter to migrate throughout blood and lymphatic vessels (13, 55) and even accessing immune-privileged sites such as the brain (56); in this way, the immune cells may contribute to the development of clinical manifestations in the newborn.

As mentioned before, cytokines can influence the replication and survival of the parasite (16–19, 41, 42, 57). It has been documented in animal models that the infection by *T. gondii* may trigger a fatal proinflammatory cytokine storm (45); in this context, its regulation, importantly mediated by IL-10 and TGF-β, has been implicated in preventing tissue destruction in several toxoplasmosis models (58–60). Despite this, the role of immunomodulatory cytokines such as TGF-β is controversial, suggesting that it inhibits inflammation, in this way diminishing tissue damage, but allowing the course of chronic infection (25). Interestingly, in support to our results, it has been demonstrated that the lack of expression of IL-2 or TGF-β1 in mice limits lymphocytes Foxp3 expression in periphery, remarking the central role of these cytokines in the induction and maintenance of regulatory T cells (Tregs) (61, 62). We found that higher production of IL-2 and TGF-β in the congenitally infected newborns was related to a mild disease outcome, probably by the induction of Tregs dampening the proinflammatory response against *T. gondii*, which as mentioned before may contribute to tissue damage, but further investigation is required. Likewise, we recently published data suggesting that TGF-β somehow protects against vertical transmission (19); thus, while a proinflammatory response is needed to respond against the parasite and limit disease in the mother, it should be finely regulated.

In conclusion, the results of this work suggest that a nonregulated increased inflammatory response is related to vertical transmission of *T. gondii* in humans (19), as well as to severity and dissemination of the parasite in congenitally infected newborns.

## Data Availability Statement

The datasets generated for this study are available on request to the corresponding author.

## Ethics Statement

The studies involving human participants were reviewed and approved by Research and Research Ethics Boards of the Instituto Nacional de Pediatría (INP), Mexico City, IRB00008064 and IRB00008065. Written informed consent to participate in this study was provided by the participants' legal guardian/next of kin.

## Author Contributions

DC, RF-D, and CL-C conceived the study design and wrote down the large project in which this study was nested. RF-D and JM-G recruited pregnant women and clinically studied them. VG-T, CL-C, GA-E, and CB-R recruited, clinically managed the newborns, and followed them for more than 1 year of age: they also defined the clinical criteria and classified the babies according to localization and severity of the disease. FG-C, YF-G, IC-S, LO-A, and HL-P conducted lab experiments. FG-C, DC, and RG-R reviewed and analyzed the data. FG-C wrote the paper. All authors revised the manuscript and approved this version.

### Conflict of Interest

The authors declare that the research was conducted in the absence of any commercial or financial relationships that could be construed as a potential conflict of interest.

## References

[1] Gilbert R Dezateux C Newborn screening for congenital toxoplasmosis: feasible, but benefits are not established. Arch Dis Child. (2006) 91:629–31. 10.1136/adc.2006.094870 16861480 PMC2083040

[2] Hill DE Dubey JP *Toxoplasma gondii* In: Ortega YR Sterling CR editors. Foodborne Parasites. Food Microbiology and Food Safety. Cham: Springer (2018). p. 119–38.

[3] Jackson MH Hutchison WM. The prevalence and source of Toxoplasma infection in the environment. Adv Parasitol. (1989) 28:55–105. 10.1016/S0065-308X(08)60331-0 2683617

[4] Dunn D Wallon M Peyron F Petersen E Peckham C Gilbert R. Mother-to-child transmission of toxoplasmosis: risk estimates for clinical counselling. Lancet. (1999) 353:1829–33. 10.1016/S0140-6736(98)08220-8 10359407

[5] Randall LM Hunter CA. Parasite dissemination and the pathogenesis of toxoplasmosis. Eur J Microbiol Immunol. (2011) 1:3–9. 10.1556/EuJMI.1.2011.1.3 24466431 PMC3894809

[6] Lambert H Barragan A. Modelling parasite dissemination: host cell subversion and immune evasion by *Toxoplasma gondii*. Cell Microbiol. (2010) 12:292–300. 10.1111/j.1462-5822.2009.01417.x 19995386

[7] Weiss LM Dubey JP. Toxoplasmosis: a history of clinical observations. Int J Parasitol. (2009) 39:895–901. 10.1016/j.ijpara.2009.02.004 19217908 PMC2704023

[8] Arce-Estrada GE Gomez-Toscano V Cedillo-Pelaez C Sesman-Bernal AL Bosch-Canto V Mayorga-Butron JL. Report of an unsual case of anophthalmia and craniofacial cleft in a newborn with *Toxoplasma gondii* congenital infection. BMC Infect Dis. (2017) 17:1–5. 10.1186/s12879-017-2565-8 28673238 PMC5494800

[9] Gómez-Toscano V Linares-López KA Arce-Estrada GE Figueroa-Damián R Barrios-Bautista DM Hernändez-Luengas L Toxoplasmosis congénita en el Valle de México. Resultados de una serie de casos. Acta Pediatr Mex. (2018) 39:321–33. 10.18233/APM39No6pp321-3331730

[10] Cañedo-Solares I Galvan-Ramirez M de L Luna-Pasten H Rodriguez Perez LR Ortiz-Alegría LB Rico-Torres CP. Congenital toxoplasmosis: specific IgG subclasses in mother/newborn pairs. Pediatr Infect Dis J. (2008) 27:469–74. 10.1097/INF.0b013e31816591df 18520342

[11] Ortiz-Alegría LB Caballero-Ortega H Cañedo-Solares I Rico-Torres CP Sahagun-Ruiz A Medina-Escutia ME. Congenital toxoplasmosis: candidate host immune genes relevant for vertical transmission and pathogenesis. Genes Immun. (2010) 11:363–73. 10.1038/gene.2010.21 20445562

[12] Rico-Torres CP Vargas-Villavicencio JA Correa D. Is *Toxoplasma gondii* type related to clinical outcome in human congenital infection? Systematic and critical review. Eur J Clin Microbiol Infect Dis. (2016) 35:1079–88. 10.1007/s10096-016-2656-2 27146878

[13] Lambert H Dellacasa-Lindberg I Barragan A. Migratory responses of leukocytes infected with *Toxoplasma gondii*. Microbes Infect. (2011) 13:96–102. 10.1016/j.micinf.2010.10.002 20951223

[14] Harker KS Ueno N Lodoen MB. *Toxoplasma gondii* dissemination: a parasite's journey through the infected host. Parasite Immunol. (2015) 37:141–9. 10.1111/pim.12163 25408224

[15] Pfaff AW Georges S Abou-Bacar A Letscher-Bru V Klein JP Mousli M. *Toxoplasma gondii* regulates ICAM-1 mediated monocyte adhesion to trophoblasts. Immunol Cell Biol. (2005) 83:483–9. 10.1111/j.1440-1711.2005.01356.x 16174097

[16] Pfaff AW Abou-Bacar A Letscher-Bru V Villard O Senegas A Mousli M. Cellular and molecular physiopathology of congenital toxoplasmosis: the dual role of IFN-gamma. Parasitology. (2007) 134:1895–902. 10.1017/S0031182007000200 17958925

[17] Abou-Bacar A Pfaff AW Georges S Letscher-Bru V Filisetti D Villard O. Role of NK cells and gamma interferon in transplacental passage of *Toxoplasma gondii* in a mouse model of primary infection. Infect Immun. (2004) 72:1397–401. 10.1128/IAI.72.3.1397-1401.2004 14977944 PMC356035

[18] Abou-Bacar A Pfaff AW Letscher-Bru V Filisetti D Rajapakse R Antoni E. Role of gamma interferon and T cells in congenital Toxoplasma transmission. Parasite Immunol. (2004) 26:315–8. 10.1111/j.0141-9838.2004.00713.x 15679627

[19] Gómez-Chávez F Cañedo-Solares I Ortiz-Alegría LB Flores-García Y Luna-Pasten H Figueroa-Damian R. Maternal immune response during pregnancy and vertical transmission in human toxoplasmosis. Front Immunol. (2019) 10:1–7. 10.3389/fimmu.2019.00285 30846989 PMC6393384

[20] Caballero-Ortega H Castillo-Cruz R Murieta S Ortiz-Alegria LB Calderon-Segura E Conde-Glez CJ. Diagnostic-test evaluation of immunoassays for anti-*Toxoplasma gondii* IgG antibodies in a random sample of Mexican population. J Infect Dev Ctries. (2014) 8:642–7. 10.3855/jidc.3858 24820469

[21] Cañedo-Solares I Ortiz-Alegría LB Figueroa-Damian R Bustos-Bahena ML González-Henkel H Calderon-Segura E. Toxoplasmosis in pregnancy: determination of IgM, IgG and avidity in filter paper-embedded blood. J Perinatol. (2009) 29:668–72. 10.1038/jp.2009.79 19554010

[22] Rico-Torres CP Figueroa-Damian R Lopez-Candiani C Macias-Aviles HA Cedillo-Pelaez C Cañedo-Solares I. Molecular diagnosis and genotyping of cases of perinatal toxoplasmosis in Mexico. Pediatr Infect Dis J. (2012) 31:411–3. 10.1097/INF.0b013e318241f564 22173138

[23] Jackson EJ A User's Guide to Principal Components. New York, NY: Wiley (1991). 592 p.

[24] Xu X Zhang J Zhan S Li Z Liu X Zhang H. TGF-β1 improving abnormal pregnancy outcomes induced by *Toxoplasma gondii* infection: regulating NKG2D/DAP10 and killer subset of decidual NK cells. Cell Immunol. (2017) 317:9–17. 10.1016/j.cellimm.2017.04.004 28438315

[25] Zare-Bidaki M Assar S Hakimi H Abdollahi SH Nosratabadi R Kennedy D TGF-beta in toxoplasmosis: friend or foe? Cytokine. (2016) 86:29–35. 10.1016/j.cyto.2016.07.002 27449809

[26] Zhao M Zhang H Liu X Jiang Y Ren L Hu X. The effect of TGF-β on Treg cells in adverse pregnancy outcome upon *Toxoplasma gondii* infection. Front Microbiol. (2017) 8:1–10. 10.3389/fmicb.2017.00901 28603517 PMC5445113

[27] Chang HR Grau GE Pechère JC. Role of TNF and IL-1 in infections with *Toxoplasma gondii*. Immunology. (1990) 69:33–7. 2107144 PMC1385716

[28] Yamamoto JH Vallochi AL Silveira C Filho JK Nussenblatt RB Cunha-Neto E. Discrimination between patients with acquired toxoplasmosis and congenital toxoplasmosis on the basis of the immune response to parasite antigens. J Infect Diseases. (2000) 181:2018–22. 10.1086/315494 10837184

[29] Safronova A Araujo A Camanzo ET Moon TJ Elliott MR Beiting DP. Alarmin S100A11 initiates a chemokine response to the human pathogen *Toxoplasma gondii*. Nat Immunol. (2019) 20:64–72. 10.1038/s41590-018-0250-8 30455460 PMC6291348

[30] Sasai M Pradipta A Yamamoto M. Host immune responses to *Toxoplasma gondii*. Int Immunol. (2018) 30:113–9. 10.1093/intimm/dxy004 29408976

[31] Fieschi C Casanova JL. The role of interleukin-12 in human infectious diseases: only a faint signature. Eur J Immunol. (2003) 33:1461–4. 10.1002/eji.200324038 12778462

[32] Sharma SD Hofflin JM Remington JS. *In vivo* recombinant interleukin 2 administration enhances survival against a lethal challenge with *Toxoplasma gondii*. J Immunol. (1985) 135:4160–3. 3877764

[33] Denkers EY Scharton-Kersten T Barbieri S Caspar P Sher A. A role for CD4+ NK1.1+ T lymphocytes as major histocompatibility complex class II independent helper cells in the generation of CD8+ effector function against intracellular infection. J Exp Med. (1996) 184:131–9. 10.1084/jem.184.1.131 8691126 PMC2192666

[34] Brière F Servet-Delprat C Bridon JM Saint-Remy JM Banchereau J. Human interleukin 10 induces naive surface immunoglobulin D+ (sIgD+) B cells to secrete IgG1 and IgG3. J Exp Med. (1994) 179:757–62. 10.1084/jem.179.2.757 8294883 PMC2191366

[35] Deenick EK Hasbold J Hodgkin PD. Switching to IgG3, IgG2b, and IgA is division linked and independent, revealing a stochastic framework for describing differentiation. J Immunol. (1999) 163:4707–14. 10528168

[36] Lourenço EV Bernardes ES Silva NM Mineo JR Panunto-Castelo A Roque-Barreira MC. Immunization with MIC1 and MIC4 induces protective immunity against *Toxoplasma gondii*. Microbes Infect. (2006) 8:1244–51. 10.1016/j.micinf.2005.11.013 16616574

[37] Correa D Cañedo-Solares I Ortiz-Alegría LB Caballero-Ortega H Rico-Torres CP. Congenital and acquired toxoplasmosis: diversity and role of antibodies in different compartments of the host. Parasite Immunol. (2007) 29:651–60. 10.1111/j.1365-3024.2007.00982.x 18042171

[38] Pernas L Ramirez R Holmes TH Montoya JG Boothroyd JC. Immune profiling of pregnant Toxoplasma-infected US and Colombia patients reveals surprising impacts of infection on peripheral blood cytokines. J Infect Dis. (2014) 210:923–31. 10.1093/infdis/jiu189 24664173 PMC4192053

[39] Castillo C Munoz L Carrillo I Liempi A Gallardo C Galanti N. *Ex vivo* infection of human placental chorionic villi explants with *Trypanosoma cruzi* and *Toxoplasma gondii* induces different Toll-like receptor expression and cytokine/chemokine profiles. Am J Reprod Immunol. (2017) 78:e12660. 10.1111/aji.12660 28328108

[40] Milian ICB Silva RJ Manzan-Martins C Barbosa BF Guirelli PM Ribeiro M. Increased *Toxoplasma gondii* intracellular proliferation in human extravillous trophoblast cells (HTR8/SVneo line) is sequentially triggered by MIF, ERK1/2, and COX-2. Front Microbiol. (2019) 10:1–13. 10.3389/fmicb.2019.00852 31068920 PMC6491458

[41] Sommerville C Richardson JM Williams RA Mottram JC Roberts CW Alexander J. Biochemical and immunological characterization of *Toxoplasma gondii* macrophage migration inhibitory factor. J Biol Chem. (2013) 288:12733–41. 10.1074/jbc.M112.419911 23443656 PMC3642319

[42] Barbosa BF Paulesu L Ietta F Bechi N Placenta R-R. Susceptibility to *Toxoplasma gondii* proliferation in BeWo human trophoblast cells is dose-dependent of macrophage migration inhibitory factor (MIF), via ERK1/2 phosphorylation and prostaglandin E2 production. Placenta. (2014) 35:152–62. 10.1016/j.placenta.2013.12.013 24433846

[43] Bhadra R Gigley JP Khan IA. The CD8 T-cell road to immunotherapy of toxoplasmosis. Immunotherapy. (2011) 3:789–801. 10.2217/imt.11.68 21668315 PMC4051159

[44] Abou-Bacar I Chiesa S Taverna P Ceccarelli R Frulio R Morandi F T cell mediated immune responses to *Toxoplasma gondii* in pregnant women with primary toxoplasmosis. Microbes Infect. (2006) 8:552–60. 10.1016/j.micinf.2005.08.008 16324868

[45] Gomes AO Oliveira DA Silva NM Effect of macrophage migration inhibitory factor (MIF) in human placental explants infected with *Toxoplasma gondii* depends on gestational age. Am J Pathol. (2011) 178:2792–801. 10.1016/j.ajpath.2011.02.005 21641401 PMC3124335

[46] Ferro EA Mineo JR Ietta F Bechi N Romagnoli R Silva DA. Macrophage migration inhibitory factor is up-regulated in human first-trimester placenta stimulated by soluble antigen of *Toxoplasma gondii*, resulting in increased monocyte adhesion on villous explants. Am J Pathol. (2008) 172:50–8. 10.2353/ajpath.2008.070432 18165264 PMC2189627

[47] Mor G Aldo P Alvero AB. The unique immunological and microbial aspects of pregnancy. Nat Rev Immunol. (2017) 17:469–82. 10.1038/nri.2017.64 28627518

[48] Remington JS McLeod R Thulliez P Desmonts G Toxoplasmosis. In: Remington JS O Klein J Wilson CB Baker CJ editors. Infectious Diseases of the Fetus and Newborn Infant. 6th ed. Amsterdam: Elsevier (2006). p. 947–1091.

[49] Lu F Huang S Kasper LH. CD4+ T cells in the pathogenesis of murine ocular toxoplasmosis. Infect Immun. (2004) 72:4966–72. 10.1128/IAI.72.9.4966-4972.2004 15321988 PMC517441

[50] Liesenfeld O Kosek J Remington JS Suzuki Y. Association of CD4+ T cell-dependent, interferon-gamma-mediated necrosis of the small intestine with genetic susceptibility of mice to peroral infection with *Toxoplasma gondii*. J Exp Med. (1996) 184:597–607. 10.1084/jem.184.2.597 8760813 PMC2192709

[51] Roberts F Mets MB Ferguson DJ O'Grady R O'Grady C Thulliez P. Histopathological features of ocular toxoplasmosis in the fetus and infant. Arch Ophthalmol. (2001) 119:51−8. 10.1001/pubs.Ophthalmol.-ISSN-0003-9950-119-1-ecs90186 11146726

[52] Machado AS Carneiro AC Bela SR Andrade GM Vasconcelos-Santos DV Januário JN. Biomarker analysis revealed distinct profiles of innate and adaptive immunity in infants with ocular lesions of congenital toxoplasmosis. Mediators Inflamm. (2014) 2014:910621. 10.1155/2014/910621 25328286 PMC4189853

[53] Carneiro AC Machado AS Bela SR Costa JG Andrade GM Vasconcelos-Santos DV. Cytokine signatures associated with early onset, active lesions and late cicatricial events of retinochoroidal commitment in infants with congenital toxoplasmosis. J Infect Dis. (2016) 213:1962–70. 10.1093/infdis/jiw041 26946460

[54] Persson EK Agnarson AM Lambert H Hitziger N Yagita H Chambers BJ. Death receptor ligation or exposure to perforin trigger rapid egress of the intracellular parasite *Toxoplasma gondii*. J Immunol. (2007) 179:8357–65. 10.4049/jimmunol.179.12.8357 18056381

[55] Bierly AL Shufesky WJ Sukhumavasi W Morelli AE Denkers EY. Dendritic cells expressing plasmacytoid marker PDCA-1 are trojan horses during *Toxoplasma gondii* infection. J Immunol. (2008) 181:8485–91. 10.4049/jimmunol.181.12.8485 19050266 PMC2626190

[56] Lachenmaier SM Deli MA Meissner M Liesenfeld O. Intracellular transport of *Toxoplasma gondii* through the blood-brain barrier. J Neuroimmunol. (2011) 232:119–30. 10.1016/j.jneuroim.2010.10.029 21106256 PMC4116595

[57] Gaddi PJ Yap GS. Cytokine regulation of immunopathology in toxoplasmosis. Immunol Cell Biol. (2007) 85:155–9. 10.1038/sj.icb.7100038 17228318

[58] Gazzinelli RT Wysocka M Hieny S Scharton-Kersten T Cheever A Kuhn R. In the absence of endogenous IL-10, mice acutely infected with *Toxoplasma gondii* succumb to a lethal immune response dependent on CD4+ T cells and accompanied by overproduction of IL-12, IFN-gamma and TNF-alpha. J Immunol. (1996) 157:798–805. 8752931

[59] Suzuki Y Sher A Yap G Park D Neyer LE Liesenfeld O. IL-10 is required for prevention of necrosis in the small intestine and mortality in both genetically resistant BALB/c and susceptible C57BL/6 mice following peroral infection with *Toxoplasma gondii*. J Immunol. (2000) 164:5375–82. 10.4049/jimmunol.164.10.5375 10799901

[60] Ohta K Yamagami S Taylor AW Streilein JW. IL-6 antagonizes TGF-beta and abolishes immune privilege in eyes with endotoxin-induced uveitis. Invest Ophthalmol Vis Sci. (2000) 41:2591–9. 10937571

[61] Horwitz DA Zheng SG Wang J Gray JD. Critical role of IL-2 and TGF-β in generation, function and stabilization of Foxp3+CD4+ Treg. Eur J Immunol. (2008) 38:912–5. 10.1002/eji.200738109 18395858

[62] Freudenberg K Lindner N Dohnke S Garbe AI Schallenberg S Kretschmer K. Critical role of TGF-β and IL-2 receptor signaling in Foxp3 induction by an inhibitor of DNA methylation. Front Immunol. (2018) 9:125. 10.3389/fimmu.2018.00125 29456534 PMC5801288

