# Supplementary material for: A Proinflammatory Immune Response Might Determine Toxoplasma gondii Vertical Transmission and Severity of Clinical Features in Congenitally Infected Newborns
Source: Front Immunol. 2020 Mar 13;11:390. doi: 10.3389/fimmu.2020.00390 (PMC7082359; doi:10.3389/fimmu.2020.00390)
Supplement: Supplementary file 1 [file Table_1.pdf]

Supplementary Table 1. Serological and clinical data of *T. gondii* infected women

Case number	Age (years)	GWD	IgG avidity	WB IgM	WB IgG	Congenital Transmission	Current pregnancy problems	History of obstetric problems
1*	33	30	Gray zone	+	+	no	-	-
2*	21	12	Gray zone	+	+	no	-	Miscarriage
3	39	34	High	+	+	no	-	Miscarriage
4*	29	12	Low	−	+	no	-	Moebius
5	36	28	High	+	−	no	Polihydroamnios	Miscarriage
6	29	13	High	+	+	no	Obesity	-
7*	36	7	Low	−	+	no	-	-
Mean	32	19		5/7	6/7			
Median	33	13						
1	21	38	High	+	+	yes	hydrocephalus oligohydramnios	-
2^	21	37	Low	−	−	yes	-	Preeclampsia
3^	17	39	Gray zone	+	+	yes	-	-
4*	-	26	Low	+	+	yes	-	Hydrops fetalis
5	33	15	High	+	+	yes	-	-
6^	27	40	Low	−	+	yes	-	-
7*^	38	12	Low	+	+	yes	-	Miscarriage, and other baby with heart malformations
8	20	40	High	+	+	yes	Oligohydramnios	-
9*	32	12	Low	−	+	yes	-	Preeclampsia
10	21	20	nd	+	−	yes	Chorionic hematoma	-
Mean	26	28		7/10	7/10			
Median	21	32						

GWD, gestational week at diagnosis. *Cases from Gómez-Chávez, et. al. 2019. ^Cases confirmed by PCR in blood.
