# Supplementary material for: A Proinflammatory Immune Response Might Determine Toxoplasma gondii Vertical Transmission and Severity of Clinical Features in Congenitally Infected Newborns
Source: Front Immunol. 2020 Mar 13;11:390. doi: 10.3389/fimmu.2020.00390 (PMC7082359; doi:10.3389/fimmu.2020.00390)
Supplement: Supplementary file 2 [file Table_2.pdf]

Supplementary Table 2. Serological and clinical data of *T. gondii* congenitally infected newborns

Case number	Gómez-Toscano et al., 2018 case number	Birth weight	Age at diagnosis (days)	WB IgM	WB IgG neoAbs	Blood PCR	Severity	Localization
1	2	3080	0	+	+	+	Mild	Local
2	8	3530	27	−	+	+	Mild	Local
3	1	2838	0	+	+	−	Mild	Local
4	9	3180	10	+	−	+	Mild	Local
5	6	2450	3	+	+	−	Mild	Disseminated
6	11	2840	0	−	+	+	Moderate/Severe	Disseminated
7	19	3000	65	−	+	+	Moderate/Severe	Disseminated
8	18	1945	28	−	+	+	Moderate/Severe	Disseminated
9	na	-	0	+	+	+	Moderate/Severe	Disseminated
10	7	1615	26	+	+	+	Moderate/Severe	Disseminated
11	10	1383	4	+	+	−	Moderate/Severe	Disseminated
